# EPPS: Efficient and Privacy-Preserving Personal Health Information Sharing in Mobile Healthcare Social Networks

**DOI:** 10.3390/s150922419

**Published:** 2015-09-03

**Authors:** Shunrong Jiang, Xiaoyan Zhu, Liangmin Wang

**Affiliations:** 1 National Key Laboratory of Integrated Services Networks, Xidian University, Xi’an 710071, China; E-Mail: jsywow@gmail.com; 2 The Department of Internet of Things, Jiangsu University, Zhenjiang 212013, China; E-Mail: wanglm@ujs.edu.cn

**Keywords:** privacy, attribute-based encryption, bloom filter, mobile healthcare social networks

## Abstract

Mobile healthcare social networks (MHSNs) have emerged as a promising next-generation healthcare system, which will significantly improve the quality of life. However, there are many security and privacy concerns before personal health information (PHI) is shared with other parities. To ensure patients’ full control over their PHI, we propose a fine-grained and scalable data access control scheme based on attribute-based encryption (ABE). Besides, policies themselves for PHI sharing may be sensitive and may reveal information about underlying PHI or about data owners or recipients. In our scheme, we let each attribute contain an attribute name and its value and adopt the Bloom filter to efficiently check attributes before decryption. Thus, the data privacy and policy privacy can be preserved in our proposed scheme. Moreover, considering the fact that the computational cost grows with the complexity of the access policy and the limitation of the resource and energy in a smart phone, we outsource ABE decryption to the cloud while preventing the cloud from learning anything about the content and access policy. The security and performance analysis is carried out to demonstrate that our proposed scheme can achieve fine-grained access policies for PHI sharing in MHSNs.

## 1. Introduction

With the rapid development of sensor systems, mobile computing and wireless communication technologies, mobile healthcare social networks (MHSNs) [1,2] have attracted tremendous attention from both industry and academia. Compared to the traditional electronic healthcare system [3,4], in MHSNs, patients can walk outside, moving freely by wearing body sensors to continuously monitor their personal health information (PHI) [5]. Smartphones can then be used to aggregate the monitored PHI via Bluetooth or ZigBee and transmit PHI data to the remote healthcare center via 2G/3G/4G networks. In this way, it extends the traditional centralized e-health system to a decentralized, self-organized way that the authorized mobile patients (*i.e.*, those holding the same symptoms and constituting a social group) are allowed to search, recognize and socially interact with each other in close proximity.

In such mobile social networks, it is more likely for patients with the same health conditions to share their health conditions and medical experience for mutual support and comfort [3]. However, security and privacy concerns are the stumbling blocks that must be cleared before this could happen.

Specifically, we must address the following issues.

- Since PHI contains sensitive information, patients should be able to control the sharing of their PHI. This means that even under a random and anonymous condition, the PHI owner should decide how to encrypt his/her files and with which set of users to share the PHI [6].
- In MHSNs, it is not only the identity, but also the social attributes of a patient that are sensitive and private. By tracking the identities of patients, an adversary may learn the identities of both the source who generated PHI packets and the intermediate patients who share/forward PHI packets. Besides, the social attributes can be inferred from the underlying PHI, the PHI owner or the PHI recipients. Therefore, both the identity and social attributes should be protected [7].
- Health-related information may be critical to patients’ lives. Good advice from a well-known doctor can be used to improve a patient’s health condition significantly, while inappropriate instruction from an unqualified doctor may put the lives of patients in danger. Therefore, the receiver should ensure/verify the correctness of the PHI used for diagnosis [8].
- Since smartphones are used not only for healthcare monitoring, but also for other applications, we should consider the cost and efficiency of the privacy-preserving scheme. Thus, the privacy-preserving PHI sharing scheme should be efficient and time-saving [9].

In this paper, we propose a patient-centric secure and privacy-preserving PHI sharing scheme for MHSNs. In order to achieve patient-centric control and fine-grained access control of PHI sharing, we adopt attribute-based encryption (ABE) as the main encryption primitive for healthcare [3,6,8,10,11,12,13,14]. Although ABE schemes can be directly applied to design secure access control, they usually use the social attributes to construct the access policy, which is sensitive, as well. Thus, to protect the sensitive social attributes of patients and to achieve efficient and anonymous ABE (Anonymous ABE means hidden the access policy for ABE), each attribute consists of two parts: a coarse-grained attribute name and its fine-grained value. We use the coarse-grained attribute name to construct the access policy while protecting the fine-grained and sensitive attribute values from others. However, to hide the access policy in ABE [7,15,16,17,18] and to realize the fine-grained access control in resource-limited smartphones [19,20], important issues, such as efficient decryption without repeating decryption attempts and energy saving, are nontrivial to address.

### 1.1. Related Work

The related work can be divided in two categories: (1) secure and private health information sharing based on ABE; and (2) the hidden access policies and outsourced decryption for ABE.

#### 1.1.1. Secure and Private Health Information Sharing Based on ABE

Ibraimi *et al.* [10] proposed a multi-authority ciphertext-policy (CP)-ABE scheme to protect EMRS. In their scheme, everyone can download the encrypted data, but only authorized users from the social domain (e.g., family, friends or fellow patients) or authorized users from the professional domain (e.g., doctors or nurses) can decrypt it. Narayan *et al.* [11] proposed an attribute-based infrastructure for EHR systems, where each patient’s EHR files were encrypted using a broadcast variant of CP-ABE. Their scheme supported direct revocation of user access without re-encrypting the data by using broadcast encryption techniques. Li *et al.* [3] proposed a novel framework to achieve patient-centric and fine-grained data access control with a cloud computing environment by adopting multi-authority attribute-based encryption (MA-ABE). A refined version of [3] has been given in [6], which extended the usage of MA-ABE in the public domain and offered a revocable MA-ABE scheme. Akinyele *et al.* [12] designed and implemented self-protecting electronic medical records (EMRs) based on ABE on mobile devices. They used ABE to achieve fine-grained access control for different items of EMRs, which can either be stored on cloud servers or mobile devices, so that the EMR could be accessed when the health provider is offline. Huang *et al.* [13] proposed a novel mobile cloud data processing framework. In their proposed framework, each mobile device was treated as a service node (SN), which was mapped to one or more extended semi-shadow images (ESSIs) in the cloud. Then, the mobile device can outsource its computing and storage services to its corresponding ESSI and secure storage (SS). To protect the privacy of outsourced data, they used identity-based signature schemes and attribute-based encryption schemes to achieve authentication and data access control, respectively. Liang *et al.* [8] proposed two attribute-oriented authentication and transmission schemes for secure and privacy-preserving health information sharing in health social networks (HSNs). The attribute-oriented authentication scheme can achieve sensitive attributes anonymity, and the attribute-oriented transmission scheme can enable fine-grained PHI sharing by using ABE. Liu *et al.* proposed [14] a new approach for fine-grained access control and secure sharing of signcrypted data for PHR in cloud computing scenarios. They named the proposed algorithm ciphertext-policy attribute-based signcryption (CP-ABSC), which combined the merits of digital signature and encryption to provide confidentiality, authenticity, unforgeability, anonymity and collusion resistance. Finally, we list the differences of related works in Table 1.

Different from these existing schemes, we focus on realizing access policy hidden in ABE and efficient decryption on the resource-constrained mobile devices, which outsource the most time-consuming decryption to the cloud.

**Table 1:** Comparison of related works.

	Multi-Authority	Broadcast	Cloud Computing	Mobile Device
[10]	*√*			
[11]		*√*		
[3]	*√*		*√*	
[12]				*√*
[13]			*√*	*√*
[8]				*√*
[14]			*√*	

#### 1.1.2. Hidden Access Policies and Outsourced Decryption for ABE

ABE was introduced by Sahai and Waters [21] to enable a public key based on one-to-many encryption, and a scalable and fine-grained access control system was realized. There are two kinds of ABE schemes: key-policy ABE (KP-ABE) and ciphertext-policy ABE (CP-ABE) schemes. Both achieve access control over encrypted data using access policies and ascribed attributes associated with private keys and ciphertexts. Although CP-ABE can realize flexible and fine-grained access control, some disadvantages of CP-ABE also were discovered, as we discuss in the following.

In traditional CP-ABE schemes, an access structure is sent along with a ciphertext explicitly. Therefore, anyone who obtains the ciphertext is able to know the associated access structure. However, it is not only the data, but also the policies for sharing the data that are sensitive. To hide the access structure, Nishide *et al.* [15] proposed their scheme, where the admitted access structures are expressed as AND gates on multi-valued attributes with wildcards. Following their work, Li *et al.* [16] studied the problem of user accountability. Recently, Lai *et al.* [17] proposed a fully-secure CP-ABE scheme with partially-hidden access structures. However, their scheme only supports restricted access structures as in [15,16]. In [7], Lai *et al.* first proposed a new model for CP-ABE with partial hidden access structures in which each attribute consists of two parts: an attribute name and the corresponding value. They tried to hide the specific attribute values of the access structure. However, to hide the access policy [7,15,16,17], a user knows whether the attributes and the policy match or not only after repeating decryption attempts. Moreover, the computational overhead of each decryption is high, since the computational cost grows with the complexity of the access policy, which usually requires many pairing computations in most of the existing ABE schemes. As a result, this direct decryption method in anonymous ABE schemes will suffer a drawback of efficiency. To overcome this problem, Zhang *et al.* [18] proposed their match-then-decrypt into the decryption of their anonymous ABE, in which a matching phase was added before the decryption phase. However, their scheme has the following disadvantages: (1) the access policy only supports AND gate; and (2) the access policy only supports all attributes in the universe. These two disadvantages make the scheme in [18] unattractive in practice. Finally, we list the computational overhead for each scheme for repeating decryption attempts in Table 2 where Mul is denoted as the time to perform one point multiplication over $G_{1}$, Pair is the time to execute a pairing operation and *n* is the number of attributes.

**Table 2:** The computational overhead for repeating decryption.

	Computation Overhead
[15]	(n+1)Pair+1Mul
[16]	>4nPair+nMul
[17]	(n+1)Pair+1Mul
[18]	3Pair+(2n+3)Mul

To realize the fine-grained access control, the access policy in CP-ABE is complex. This leads to the computational burden in the decryption phase, since its computational overhead grows with the number of attributes specified in the access policy. The drawback appears to be more serious for resource-constrained equipment, such as mobile devices and sensors. Green *et al.* [19] proposed a solution to this problem by introducing the notion of ABE with outsourced decryption, which largely eliminated the decryption overhead for users. However, the scheme provided no guarantee on the correctness of the transformation done by the cloud server. In the cloud computing setting, cloud service providers may have strong financial incentives to return incorrect answers, if such answers require less work and are unlikely to be detected by users. To achieve the verifiability of outsourced CP-ABE decryption, Lai *et al.* [22] proposed their scheme. However, they did not consider hiding attributes of the access policy.

### 1.2. Contributions

The main contributions of this paper are listed below:

- We propose an efficient and privacy-preserving PHI sharing scheme for smartphones in MHSNs by using anonymous ABE to achieve user-centric and fine-grained access control.
- To avoid sensitive attribute leakage and unnecessary repeating decryption attempts in anonymous ABE, we use the Bloom filter to realize partial access policy hidden from intended users and conduct the access policy matching before decryption.
- Considering the resource and energy limitation of smartphones, we outsource ABE decryption to the cloud without leaking private information and verify the correctness of partial decryption.
- Security analysis and performance evaluation have been carried out and show that the proposed PHI sharing scheme can effectively protect patients’ privacy and is suitable for resource-limited smartphones.

The rest of this paper is organized as follows: Section 2 presents the system model and preliminaries. We describe the detail of our efficient and privacy-preserving PHI sharing scheme in Section 3. We conduct the security and privacy analysis in Section 4. Section 5 provides the performance evaluation of our scheme. Finally, Section 6 concludes this paper.

## 2. System Model and Preliminaries

In this section, we present the system model and preliminaries in our scheme.

### 2.1. System Model

**Figure 1:**
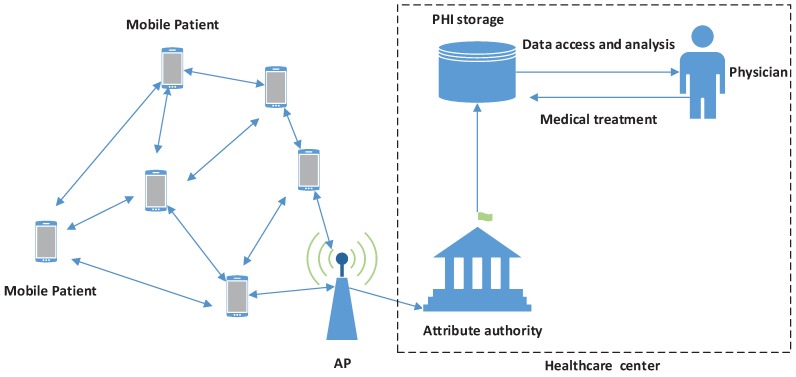
The system model for mobile healthcare social networks (MHSNs).

We give the system model in Figure 1. According to [1], the mobile patients possessing the same symptoms *(i.e.*, suffering from the same disease) and/or living in the neighborhood can constitute a social group. Based on the premise, we first divide the system into different groups, each of which has an attribute authority (AA). The AA is a trustable and powerful entity located at the healthcare center and is mainly responsible for managing the group, such as initializing the system, equipping proper body sensor nodes (BSNs) and key materials to medical users. BSNs and the smartphone periodically collect PHI and report to the AA. Patients belonging to the same AA can securely share the PHI or their own medical experience with each other using the system parameters of the AA and the secure information.

Without loss of generality, we will not consider the possibility of sharing a secret with others, because this type of active attack cannot be prevented in almost all security systems.

### 2.2. Security Requirements

1. Patient-centric access control: This means a patient can decide which set of users can gain access to his or her own PHI in MHSNs.
2. Fine-grained access control: Unauthorized users who do not possess enough attributes to meet the access policy cannot decrypt the PHI. Fine-grained access control means different users are authorized to read different sets of PHI.
3. Patient’s privacy preservation: Privacy is one of the most important concerns from a patient’s perspective. Both the identity and attributes are sensitive during the PHI sharing process and should be well protected.
4. PHI integrity, source authentication and non-repudiation: All sharing PHI should be delivered unaltered, and the origin of the messages should be authenticated by the healthcare center. To ensure non-repudiation, the patient cannot refute the validity of the PHI afterward.
5. Resistance to collusion attacks: A collusion attack is launched by multiple eavesdropping attackers. They are fully collaborative and share every secret they have to obtain confidential information, which should be prevented during the PHI sharing process.

### 2.3. Bilinear Pairing

Let G be a multiplicative cyclic group of prime order *p*, generated by element g∈G. Let $G_{T}$ be a multiplicative cyclic group of the same order *p*, such that there exists a pairing *e*: $G\times G\to G_{T}$ with the following properties [23]:

- Bilinearity: $e\left(P^{a},Q^{b}\right)=e{\left(P,Q\right)}^{ab}$ for all P,Q∈G and $a,b\in Z_{n}$;
- Non-degeneracy: e(g,g)≠1;
- Computability: For all P,Q∈G, e(P,Q) is efficiently computable.

### 2.4. Linear Secret Sharing Scheme

**Definition 1.** *(Linear secret-sharing scheme, LSSS [24]). A secret sharing scheme A over a set of parties P is called linear (over $Z_{p}$) if:*

1. the shares for each part form a vector over $Z_{p}$;
2. *there exists a matrix **M** with l rows and n columns called the share-generating matrix for A. For all i=1,···,l, the i-th row of **M** is labeled by a party ρ(i) (ρ is a function from {1,···,l} to P). When we consider the column vector $v=(s,r_{2},\cdot \cdot \cdot ,r_{n})$, where $s\in Z_{p}$ is the secret to be shared and $r_{2},\cdot \cdot \cdot ,r_{n}\in Z_{p}$ are randomly chosen, then **M**v is the vector of l shares of the secret s according to A. The share ${\left(Mv\right)}_{i}$ belongs to party ρ(i)*.

**Figure 2:**
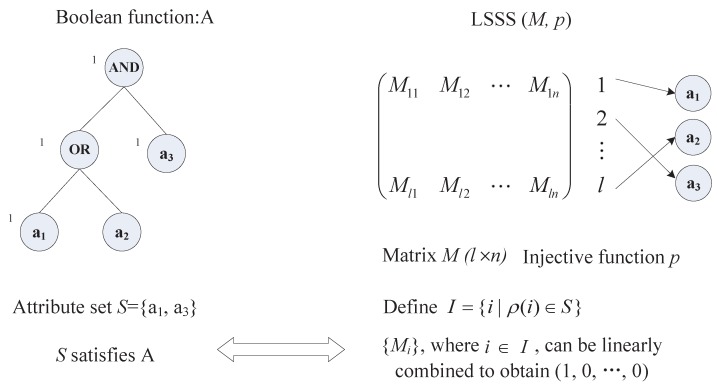
An attribute set satisfying a linear secret-sharing scheme (LSSS).

It is shown in [24] that every linear secret-sharing scheme according to the above definition also enjoys the linear reconstruction property, defined as follows. Suppose that a linear sharing structure A = (M,ρ) can be satisfied by an attribute set *S*, as shown in Figure 2, where ***M*** is a l×n matrix and ρ is an injective function from {1,···,l} to any attribute. Let I={i|ρ(i)∈S}. Therefore, there exist constants $\left\{w_{i}\in Z_{p}\right\}$, such that $\sum_{i\in I}w_{i}M_{i}$ = (1,0,···,0), where $M_{i}$ is the *i*-th row of matrix ***M***. On the other hand, if *S* does not satisfy A, those constants $\left\{w_{i}\right\}$ do not exist. These constants $\left\{w_{i}\right\}$ can be found in the time polynomial with the size of the matrix ***M*** [24]. Moreover, the inner product $Mv^{T}={\left(\lambda_{1},\cdot \cdot \cdot ,\lambda_{l}\right)}^{T}$ can be regarded as the linear secret sharing. Given an attribute set *S* and its corresponding rows I={i|ρ(i)∈S} in the matrix ***M***, finding $\left\{w_{i}\in Z_{p}\right\}$ satisfying $\sum_{i\in I}w_{i}\cdot \lambda_{i}=s$ is called linear secret reconstruction.

### 2.5. Bloom Filter

A Bloom filter is a simple space-efficient randomized data structure for representing a set *S* in order to support membership queries [25]. From the point of data storage, it is a bit array with size *m*. Bloom filters have two operations: add(x) and query(x), where *x* is an element. The add operation consists of hashing an element with several hash functions $h_{1},\cdot \cdot \cdot ,h_{k}$, which uniformly map the element to a number, such as $h_{i}\left(x\right)=y_{i}\in \left[1:m\right]$, and setting the $y_{i}$-th bit in the array to one (initially, the array is filled with zeroes). The query operation repeats the same hashing procedure and then checks if the appropriate bits are set as one. A false positive probability *p* exists when determining whether an element *x* belongs to a set or not because of the hash collision property. We can calculate *p* as follows [25]:

(1) $$\begin{array}{c} p=\left(1-{\left(1-\frac{1}{m}\right)}^{kn}k\right)\approx {\left(1-e^{\frac{-kn}{m}}\right)}^{k}, \end{array}$$

where *n* is the number of elements in set *S*. It is obvious that when $k=\left(ln2\right)\frac{m}{n}$, the false positive probability *p* is minimal, *i.e.*, ${\left(0.6185\right)}^{\frac{m}{n}}$.

### 2.6. Hidden Access Policy for ABE With Efficient Decryption by the Bloom Filter

**Figure 3:**
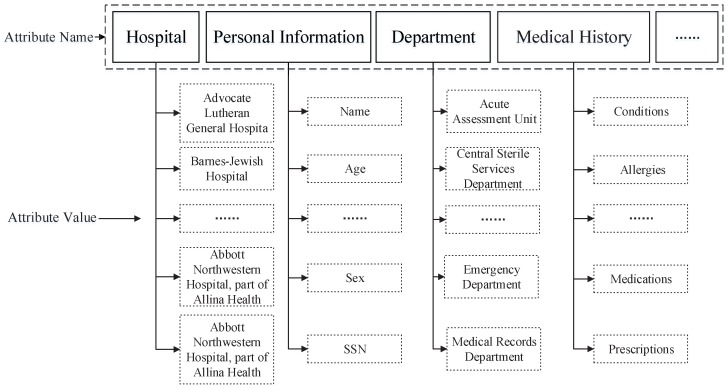
The attribute names and values of personal health information (PHI).

In our construction, each attribute includes two parts: the attribute name and its value according to [7], as Figure 3 shows. Without loss of generality, we assume that there are *n* categories of attributes (attribute name) in the universe of attributes U = $\left\{an_{1},an_{2},\cdot \cdot \cdot ,an_{n}\right\}$, and each attribute has multiple attribute values, where $S_{i}$ = $\left\{v_{i,1},v_{i,2},\cdot \cdot \cdot ,v_{i,n_{i}}\right\}$ is the multiple values set for $an_{i}$ and $|S_{i}|$ = $n_{i}$. A user’s $U_{A}$ attribute set has *n* attribute values, each of which belongs to a different attribute name and is expressed as $A_{U_{A}}$ = ($s_{1},s_{2},\cdot \cdot \cdot ,s_{n}$), where $s_{i}=v_{i,t}$ is the value of attribute *i* for a user.

We express an access policy as (***M***, ρ, Γ), where ***M*** is an l×n share-generation matrix, ρ is a map from each row of ***M*** to an attribute name (*i.e.*, ρ is a function from {1,···,l} to {1,···,n}), Γ can be parsed as ($t_{\rho (1)},\cdot \cdot \cdot ,t_{\rho (l)}$) and is the value of attribute ρ(i) specified by the access policy.

**Figure 4:**
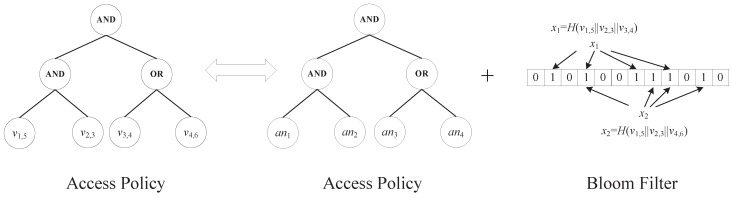
An access policy consisting of attribute values can be expressed by an access policy consisting of attribute names and a Bloom filter consisting of attribute values.

Using our notations, user $U_{A}$’s attribute set $A_{U_{A}}=\left(s_{1},\cdot \cdot \cdot ,s_{n}\right)$ satisfies an access policy (***M***, ρ, Γ) if and only if there exist I⊆{1,···,l} and constants ${\left\{w_{i}\right\}}_{i\in I}$, such that:

(2) $$\Sigma_{i\in I}w_{i}M_{i}=\left(1,0,\cdot \cdot \cdot ,0\right)\;\mathrm{and}\;s_{\rho (i)}=t_{\rho (i)}\;\mathrm{for}\;\forall i\in I,$$

where $M_{i}$ is the *i*-th row of ***M***. In our construction to be presented below, the specific attribute values (*i.e.*, Γ) of an access policy (***M***, ρ, Γ) are hidden, while other information about the access policy (*i.e.*, (***M***, ρ)) is sent along with the ciphertext explicitly. As Figure 4 shows, we do not give the LSSS of the access policy, which can be found in Figure 2.

For the decryption process, even though attribute names of the receiver meet the access policy (***M***, ρ), it cannot ensure that he/she decrypts the ciphertext and gets the plaintext. Since each attribute contains many values, the receiver only can check all possible attribute values that satisfy $\sum_{i\in I}w_{i}M_{i}=\left(1,0,\cdot \cdot \cdot ,0\right)$ where I⊆{1,···,l} to decrypt the ciphertext and get the plaintext [7]. It is obvious that this will cause excessive unnecessary computational overhead. To overcome this drawback, we adopt the Bloom filter BF to load all hash values $h(\Gamma_{I})$ where $\Gamma_{I}=\left(t_{\rho (1)},\cdot \cdot \cdot ,t_{\rho (i)}\right)$ for I⊆{1,···,l} satisfies Equation (2) and h(·) is a hash function. During decryption, the receiver extracts the corresponding attribute values according to (***M***, ρ), and computes $h(\Gamma_{I})$ for all $\sum_{i\in I}w_{i}M_{i}=\left(1,0,\cdot \cdot \cdot ,0\right)$. Then, the receiver verifies $\left[BF\left[h_{1}\left(h\left(\Gamma_{I}\right)\right)\right]=1\right]\wedge \left[BF\left[h_{2}\left(h\left(\Gamma_{I}\right)\right)\right]=1\right]\cdot \cdot \cdot \wedge \left[BF\left[h_{k}\left(h\left(\Gamma_{I}\right)\right)\right]=1\right]$ to decrypt the ciphertext, where $h_{1}\left(\cdot \right),h_{2}\left(\cdot \right),\cdot \cdot \cdot ,h_{k}\left(\cdot \right)$ are hash functions. If it is satisfied, then the corresponding secret keys can be used to decrypt the ciphertext. There are two decryption approaches that can be adopted to get the plaintext: (1) decrypting on one’s own smartphone; and (2) outsourcing the decryption to the cloud. For decryption on one’s own smartphone, the receiver directly decrypts the ciphertext by using the corresponding secret keys; while for outsourcing decryption, the receiver outsources most decryption work to the cloud without leaking the plaintext. After getting the partial decryption result from the cloud, the receiver is able to get the plaintext with one simple exponentiation operation.

## 3. Proposed Schemes

In this section, we will introduce our PHI sharing scheme. First, we give an overview of our scheme with different scenarios. Then, we will describe the details of our scheme.

### 3.1. Overview

The main goal of our scheme is to provide patient-centric secure and privacy-preserving PHI sharing in MHSNs. First, the system can be divided into different groups (e.g., by hospitals), each of which has an AA to manage its patients and implements the PHI sharing process. After the group is formed, the AA initializes the PHI sharing process by generating system parameters. The patients register with the AA and get the attribute-based secret keys from the AA according to their profiles.

Then, patients can launch the PHI sharing process. Before sharing PHI, the identity and group should be verified, which ensures that the validity of a passing-by person has the same medical software for PHI sharing or communication. In the PHI sharing phase, the patient-centric fine-grained PHI access control is achieved by using ABE, and the receiver can get the PHI when his/her attribute values meet the hidden access policy. Furthermore, in view of the computational cost growing with the complexity of the access policy, resource, as well as energy limitation of smartphones, we shift the heavy computational overhead to the cloud.

Considering the poor performance for repeating decryption attempts of the existing hidden access policy in ABE [7,15,16,17], we adopt the match-then-decrypt in decryption in anonymous ABE, in which a matching phase based on the Bloom filter is added before the decryption phase.

### 3.2. Hidden Access Policy Based on the Bloom Filter

We introduce EPPS in three steps: first, in the initialization phase, the AA generates the public parameters and secret keys for the users; second, an authentication scheme is presented to enable users to authenticate identity and group while preserving their privacy; third, a fine-grained PHI sharing scheme is proposed to allow users to achieve PHI sharing according to the hidden access policy. Moreover, an outsourced decryption for sharing PHI is also proposed. Due to space limitations, we omit the description of the authentication phase, which can be found in [9,20]. In the following, we present the details of each step.

#### 3.2.1. System Initialization

The AA is in charge of the whole system bootstrap. As is commonly done, the AA first generates the public parameters ($p,G,G_{T},e$) where G and $G_{T}$ are multiplicative cyclic groups of prime order *p*. The attribute universe is described as $U\subseteq Z_{p}^{*}$. For each attribute i∈U, the AA chooses a random value $u_{i}\in Z_{p}^{*}$. Next, it picks g,u∈G and α, $a\in Z_{p}^{*}$ uniformly at random. Finally, it chooses a collision-resistant hash function H(). The public parameters PP are published as:

(3) $$\mathrm{PP}=(G,G_{T},e,g,g^{a},e{\left(g,g\right)}^{\alpha },H\left(\right),T_{i}=g^{u_{i}}\forall i),$$

and the master secret key MSK is:

(4) MSK=α.

When user $U_{A}$ wants to join MHSNs, he/she should register with an AA. $U_{A}$ sends his/her personal health profile containing different attributes and symptom characteristics to the AA. After verifying the validity of $U_{A}$’s health profile, the AA chooses the corresponding body sensor nodes to establish personal BSNs and then installs the necessary medical software in the smartphone.

To prevent the privacy of users during the PHI sharing process, the AA also generates a family of unlinkable pseudo identities $PID_{U_{A}}$={$pid_{U_{A}}^{1}$, $pid_{U_{A}}^{2}$, ···}, where each $pid_{U_{A}}^{j}$ is computed by $pid_{U_{A}}^{j}$=$Enc_{mk}(U_{A}\left|\right|r_{U_{A}}^{j})$, where $r_{U_{A}}^{j}$ is a random number and mk is the master key of the AA, and the corresponding private key is $sk_{U_{A}}^{j}$=$mk\cdot H(pid_{U_{A}}^{j})$.

For $U_{A}$, who has attribute values $A_{U_{A}}=\left(s_{1},\cdot \cdot \cdot ,s_{n}\right)$, the AA generates the security key according to $A_{U_{A}}$. The AA randomly chooses $t\in Z_{p}^{*}$ and computes the secret key ${\mathrm{SK}}_{U_{A}}$=($K,K^{\prime },{K_{i}}_{\left\{1\leq i\leq n\right\}}$) as:

(5) $$\begin{array}{c} K=g^{\alpha }g^{at},K^{\prime }=g^{t},K_{i}=T_{i}^{t}. \end{array}$$

#### 3.2.2. PHI Sharing

For user $U_{A}$, who wants to share his/her PHI, he/she should execute Algorithm 1 to realize fine-grained access control of PHI. Here, ***M*** is an l×n matrix, ρ is a map from each row $M_{i}$ of ***M*** to an attribute name, $\Gamma =\left(t_{\rho (1)},\cdot \cdot \cdot ,t_{\rho (l)}\right)\in Z_{p}^{l}$ and $C_{2}=Sig_{sk_{U_{A}}^{j}}\left(C_{1}\right)$ is the signature of $C_{1}$ with $sk_{U_{A}}^{j}$.

**Algorithm 1** Encrypt messages by $U_{A}$.**Require:** Shared PHI message MES and (A,ρ,Γ).1: $U_{A}$ chooses a random vector $v=\left(s,r_{2},\cdot \cdot \cdot ,r_{n}\right)\in Z_{p}^{n}$.2: $U_{A}$ also chooses $r_{x}\in Z_{p}$, for 1≤x≤l.3: $U_{A}$ computes:
$C_{1}=\left\{\begin{array}{c} \hat{C}=u^{H(\mathrm{MES})};\\ \tilde{C_{1}}=\mathrm{MES}\cdot e{\left(g,g\right)}^{\alpha s};\\ C_{1}^{\prime }=g^{s};\\ C_{1,x}=g^{aM_{i}\cdot v}{\left(T_{\rho (x)}^{H(t_{\rho }\left(x\right))}\right)}^{-r_{x}};\\ D_{1,x}=g^{r_{x}}; \end{array}\right.$4: $U_{A}$ computes $C_{2}=Sig_{sk_{U_{A}}^{j}}\left(C_{1}\right)$.5: For each I⊆{1,···,l}, which satisfies $\sum_{i\in I}w_{i}M_{i}=\left(1,0,\cdot \cdot \cdot ,0\right)$, extract the corresponding attribute values $\Gamma_{I}=\left(t_{\rho (1)},\cdot \cdot \cdot ,t_{\rho (i)}\right)$.6: Compute
$H(\Gamma_{I})$ for all I⊆{1,···,l}.7: Construct the Bloom filter
BF by using $H(\Gamma_{I})$.8: $U_{A}$ broadcasts message $C=<BF,\left(M,\rho \right),C_{1},C_{2}>$.

When $U_{B}$ receives message *C* and the corresponding Bloom filter, the receiver first verifies the signature $C_{2}$. If the signature is valid, then the receiver will decrypt $C_{1}$ according to ${\mathrm{SK}}_{U_{B}}$ and $S=(s_{1},\cdot \cdot \cdot ,s_{n})$. During the process of decryption, the receiver extracts the corresponding attribute values according to (***M***, ρ) and computes $H(\Gamma_{I})$ for all $\sum_{i\in I}w_{i}M_{i}=\left(1,0,\cdot \cdot \cdot ,0\right)$. Then, the receiver verifies $\left[BF\left[h_{1}\left(h\left(\Gamma_{I}\right)\right)\right]=1\right]\wedge \left[BF\left[h_{2}\left(h\left(\Gamma_{I}\right)\right)\right]=1\right]\cdot \cdot \cdot \wedge \left[BF\left[h_{k}\left(h\left(\Gamma_{I}\right)\right)\right]=1\right]$. If it is satisfied, two decryption approaches can be adopted to get the plaintext: (1) decrypting on one’s own smartphone; and (2) outsourcing the decryption to the cloud. We describe them as follows:

(1) Decrypting on one’s own smartphone: The receiver computes:
  (6) $$\begin{array}{cc} \frac{e(C_{1}^{\prime },K)}{\left(\prod_{i\in I}{\left(e\left(C_{1,i},K^{\prime }\right)\cdot e\left(D_{1,i},K_{\rho (i)}\right)\right)}^{w_{i}}\right)}= & \frac{e(g^{s},\left(g^{\alpha }g^{at}\right))}{\left(\prod_{i\in I}{\left(e\left(g^{aM_{i}\cdot v}T_{\rho (x)}^{-r_{x}},g^{t}\right)\cdot e\left(g^{r_{x}},{T_{\rho (x)}}^{t}\right)\right)}^{w_{i}}\right)}\\ = & \frac{e{\left(g,g\right)}^{\alpha s}e{\left(g,g\right)}^{as}}{\prod_{i\in I}e{\left(g,g\right)}^{aM_{i}\cdot v\cdot w_{i}}}=e{\left(g,g\right)}^{\alpha s}=C_{1}. \end{array}$$
  Then, the receiver computes:
  (7) $$\begin{array}{cc} & \frac{\tilde{C_{1}}}{C_{1}}=\frac{\mathrm{MES}\cdot e{\left(g,g\right)}^{\alpha s}}{e{\left(g,g\right)}^{\alpha s}}=\mathrm{MES}. \end{array}$$
(2) Outsourcing the decryption to the cloud: In this situation, the smartphone first generates the transformation key ${\mathrm{TK}}_{U_{B}}$ for the cloud before it outsources the decryption and then obtains ${\mathrm{SK}}_{U_{B}}^{\prime }$. To generate ${\mathrm{TK}}_{U_{B}}$ and ${\mathrm{SK}}_{U_{B}}^{\prime }$, $U_{B}$ chooses a random value $z\in Z_{p}$ and computes the transformation key ${\mathrm{TK}}_{U_{B}}$ as:
  (8) $$\begin{array}{c} {\mathrm{TK}}_{U_{B}}=\left({\left(g^{\alpha }g^{at}\right)}^{1/z},{\left(g^{t}\right)}^{1/z},{\left(T_{i}^{t}\right)}^{1/z}\right), \end{array}$$
  and outputs the secret key ${\mathrm{SK}}_{U_{B}}^{\prime }$ = *z*. Note that we let the user himself generate the transformation key, which is more flexible. Then, the receiver sends $\tilde{C_{1}},C_{1}^{\prime },{C_{1,x},D_{1,x}}_{\left\{1\leq x\leq l\right\}}$ and ${\mathrm{TK}}_{U_{B}}$ to the cloud for outsourced decryption.

The cloud computes:

(9) $$\begin{array}{c} \frac{e(C_{1}^{\prime },K^{1/z})}{\left(\prod_{i\in I}{\left(e\left(C_{1,i},K^{\prime 1/z}\right)\cdot e\left(D_{1,i},K_{\rho (i)}^{1/z}\right)\right)}^{w_{i}}\right)}=\frac{e{\left(g,g\right)}^{\alpha s/z}e{\left(g,g\right)}^{ats/z}}{\left(\prod_{i\in I}e{\left(g,g\right)}^{atM_{i}\cdot v\cdot w_{i}/z}\right)}=e{\left(g,g\right)}^{\alpha s/z}=C^{\prime }. \end{array}$$

then sends $C^{\prime }$ to the user $U_{B}$. After receiving $C^{\prime }$, $U_{B}$ computes:

(10) $$\begin{array}{cc} & \frac{\tilde{C_{1}}}{{C^{\prime }}^{z}}=\frac{\mathrm{MES}\cdot e{\left(g,g\right)}^{\alpha s}}{e{\left(g,g\right)}^{(\alpha s/z)z}}=\frac{\mathrm{MES}\cdot e{\left(g,g\right)}^{\alpha s}}{e{\left(g,g\right)}^{\alpha s}}=\mathrm{MES}. \end{array}$$

Finally, if $u^{H(\mathrm{MES})}=\hat{C}$, $U_{B}$ outputs the message MES; otherwise, $U_{B}$ outputs ⊥.

### 3.3. Extension

Obviously, our EPPS cannot resist the collusion attack on the access policy. To resist this attack, we add some additional requirements for our scheme under the same situation as in [18]. We describe the changes of our scheme as follows: each access policy is expressed with *n* attribute values in the form of “($v_{1,i}$ and $v_{2,j}$ and ··· and $v_{n,k}$)”, where $v_{i,j}$ is the *j*-th value of the *i*-th attribute name. If a user belongs to several groups, he/she can have several forms of “($v_{1,i}$ and $v_{2,j}$ and ··· and $v_{n,k}$)”. Then, during the key generation process, the AA will issue the private keys according to the number of groups to which the user belongs and use its secret key to sign the hash value of his/her group attribute values as $g_{i}$=$Sig_{sk_{AA}}(h(v_{1,i}$, $v_{2,j}$, ···, $v_{n,k}$)). During the PHI sharing process, $U_{A}$, who wants to share the PHI, does not need the access policy A, and the Bloom filter is generated as $h_{1}(h(\Gamma_{I}\left|\right|g_{i}))$, $h_{2}(h(\Gamma_{I}\left|\right|g_{i}))$, ···, $h_{k}(h(\Gamma_{I}\left|\right|g_{i}))$.

## 4. Security Analysis

Patient-centric access control: It is obvious that after the system initialization, $U_{A}$ gets the security key according to his/her attribute values. During the PHI sharing process, $U_{A}$ encrypts the shared PHI by the security key according to the construction access policy under ABE. Thus, the user can completely decide with whom to share the PHI in MHSNs, which fulfills the purpose of patient-centric access control.

Fine-grained access control: During the PHI sharing, $U_{A}$ adopts ABE to encrypt the shared PHI, which realizes fine-grained access control by constructing the access policy. When other users receive the encrypted shared PHI, only the authorized users who satisfy the access policy can decrypt the PHI. In this way, EPPS can achieve fine-grained access control.

Patient’s privacy preservation: The proposed scheme can ensure users’ identity privacy. During the PHI sharing process, we use a set of unlinkable pseudo identities instead of their real identities. These pseudo identities $pid_{U_{A}}^{j}$=$Enc_{mk}(U_{A}\left|\right|r_{U_{A}}^{j})$ are generated from the identity of $U_{A}$, a random number $r_{U_{A}}^{j}$ and the master key *s* of the AA. Without the master key *s* or $r_{U_{A}}^{j}$, it is impossible to infer the real identity of $U_{A}$. We use the coarse-grained attribute name to construct the access policy, while protecting the fine-grained and sensitive attribute values from others. During the whole PHI sharing process, others only know the attribute name; thus, the specific attribute values are protected. In this way, both the identity and attribute privacy are protected during the PHI sharing process.

PHI integrity, source authentication and non-repudiation: For message *C*, we use the IBE-based signature $C_{2}$ to ensure the integrity, source authentication and non-repudiation. Since the correctness of health-related information is very critical in protecting patients’ privacy, we use the $\hat{C}=u^{H(\mathrm{MES})}$ to verify the decrypted result. This is significantly important for outsourced decryption, since we cannot ensure the correctness of the cloud decryption.

Resistance to collusion attack: There are two important contents that should be protected against collusion attacks: attribute values and the shared PHI. For attribute values, we hide the decrypted attribute values by hashing attribute values, then hide them in the Bloom filter. For attackers, they are fully collaborative and share every secret they have obtained during decryption. Attribute values are hidden by the hash function, which is easy to compute, but hard to invert. Moreover, we use the Bloom filter to prevent attackers from getting the hash values. By combining the hash function and Bloom filter, we can resist collusion attacks. For the shared PHI, even if attackers have the attribute values for decryption, they cannot get the plaintext. For user $U_{A}$ with attribute values $S=(s_{1},s_{2},\cdot \cdot \cdot ,s_{n})$, the corresponding secret key is ($K=g^{\alpha }g^{at},K^{\prime }=g^{t},K_{i}=T_{i}^{t}$). For different users, *t* is different, which makes colluding attackers unable to decrypt the ciphertext. Therefore, the proposed scheme can effectively resist the collusion attacks.

## 5. Performance Analysis

In order to evaluate the practicality of our proposed EPPS, we first implement the outsourced decryption process without considering the match process on smartphones to show the efficiency of our scheme. Then, we compare the match process with existing schemes. The performance analysis is in terms of asymptotic complexity and actual implementation time. Notice that, since our EPPS focuses on sharing the PHI among smartphones, meanwhile hiding the access policy, we do not compare our scheme with other existing PHI sharing schemes [3,4,6,8].

### 5.1. The Efficiency of Outsource Process

#### 5.1.1. Asymptotic Complexity Comparison

Asymptotic complexity is measured in terms of communication overhead and computational overhead listed in Table 3. Here, |G|, $|G_{T}|$ and $|Z_{q}|$ denote the cardinalities of G, $G_{T}$ and $Z_{q}$, respectively. The Pair denotes the pairing operation; Exp denotes the exponentiation operation in G; Mul denotes the multiplication operation in G; and $Exp_{G_{T}}$ represents the exponentiation operation in $G_{T}$.

**Table 3:** The comparison of the computational overhead. ABE, attribute-based encryption.

	(${\mathrm{TK}}_{U_{B}}$)	${\mathrm{SK}}_{U_{B}}^{\prime }$	ABE Ciphertext	Cloud Decryption	Final Decryption
Size	(n+2)|G|	$|Z_{q}|$	${\left|G\right|}_{T}+2l\left|G\right|$	${\left|G\right|}_{T}+2l\left|G\right|$	${\left|G\right|}_{T}$
Computation	(n+2)Exp	0	(4Exp+2Mul)l	(2l+1)Pair+lExp	$Exp_{G_{T}}$

#### 5.1.2. Implementation

To evaluate the performance of our scheme, we implement EPPS in software based on the libfenc library [26] and JPBC library [27]. We compile the libfenc library on the VMware machine with Ubuntu 13.10 OS, 2.20 GHz Intel Core2 Duo CPU (T6600) and 1 GB RAM as the cloud server. Then, we program the final decryption process after partial decryption on an Android smartphone with 1200 MHz ARM-based HUAWEI Ascend G6 with 1 GB RAM running Android 4.3 OS by using the JPBC library. In our implementation, the bilinear map is Type A pairing (*l* = 512) with a level of 1024-bit DLOG security [28]. Note that we omit the Bloom filter checking process (We give the communication and computational overhead of this process in the match comparison section), since it only contains several hash operations, which is negligible compared to pairing operations.

The detail of the code is described as follows. Since we use the libfenc library, our implementation also adopts the key encapsulation mechanism as [19], where the ABE ciphertext is the encryption of a symmetric key $k=e{\left(g,g\right)}^{\alpha s}$, and the message is encrypted by using *k* under the AES scheme. Moreover, in our implementation, we omit components $\tilde{C_{1}}$ in $C_{1}$ by using the hash values of $e{\left(g,g\right)}^{\alpha s}$. The access policy is expressed as ($v_{1,i}$ and $v_{2,j}$ and ··· and $v_{n,k}$), where $v_{i,j}$ is the *j*-th value of the *i*-th attribute and *n* is increasing from 1–100. To assess the practicality of the outsourcing decryption, we program the final decryption process by JPBC [27] and use file input/output to simulate the communication process between the cloud and the smartphone. For each access policy, we repeat our implementation 50 times on the VMware machine and 20 times on the smartphone.

**Figure 5:**
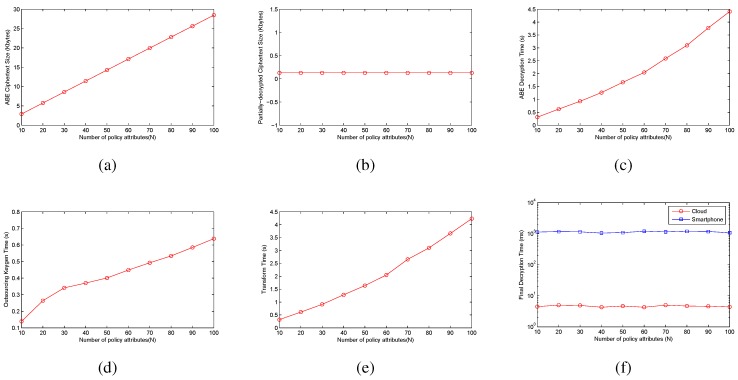
The performance of our ABE with outsourced decryption. (**a**) ABE ciphertext size; (**b**) partially-decrypted ciphertext size; (**c**) decryption time; (**d**) transformation keygen; (**e**) partial decryption time for the cloud; (**f**) final decryption time.

In Figure 5, we give the simulation results. Figure 5a,b gives the size of standard ABE ciphertext and the partially-decrypted ciphertext, respectively. Figure 5c shows the standard ABE decryption time on the VMware machine. Figure 5d indicates the time of transformation key generation. Figure 5e gives the time of transforming the ABE ciphertext. Figure 5f shows the time of decrypting the transformed ciphertext on the VMware machine and the smartphone. Notice that we do not show the standard ABE decryption time and the transformation key generation time on the smartphone, since many pairing and exponentiation operations should be executed for these two processes, which are time-consuming by JPBC [29].

As described in Figure 5a,c,e, the ciphertext size and decryption/cloud decryption time increase with the number of attributes for the ciphertext policy. Encryption under a ciphertext policy with 100 attributes results in almost a 28.5-KB file size of ABE ciphertext, and it takes about 4.5 s for the VMware machine to decrypt this ciphertext. The time for cloud decryption is almost the same as the decryption on the VMware machine, since we use the VMware machine as the cloud outsourced decryption platform, and the computation operations are the same as the VMware machine decrypting this ciphertext.

Outsourcing obviously reduces both the ciphertext size and decryption time for the partially-decrypted ciphertext. Each partially-decrypted ciphertext has a fixed 128-byte size, regardless of the number of attributes in the original ciphertext policy. Furthermore, the final decryption requires only about 4.5 ms on the VMware machine and approximate 1.1 s on the smartphone. Thus, outsourced decryption can provide a noticeable decryption time advantage for ciphertexts with a complex access policy.

Figure 5d illustrates the transformation key generation time with the number of user attributes on the VMware machine. The time is approximately linear with the number of attributes. It only takes about 0.5 s to generate transformation keys with 100 attributes. This process can be done offline.

Notice that we did not compare the decryption time of our scheme with other hidden access policy schemes [7,15,16,17], since the match processes are independent of the ABE decryption process.

### 5.2. The Efficiency of the Match Process

In this subsection, we compare our scheme with the scheme proposed by Zhang *et al.* [18], the first match-then-decrypt scheme to enhance the decryption efficiency, in terms of asymptotic complexity and actual implementation time. Both of these two schemes are under the same application condition.

#### 5.2.1. Asymptotic Complexity

Asymptotic complexity is measured in terms of communication overhead and computational overhead.

Communication overhead: The communication overhead is (5+2n)|G| bits for the scheme by Zhang *et al.* [18]. For our scheme, we assume that the number of $h(\Gamma_{I}||g_{i})$ is the same as that of the attribute name *n*, the number of elements in the Bloom filter. Let *k* = 5 denote the number of hash functions used in Bloom filter. Thus, we can get *m* = $\left\lfloor \frac{nk}{ln2}\right\rfloor =\left\lfloor \frac{5n}{ln2}\right\rfloor$. In our implementation, the bilinear map is Type A pairing (*l* = 512) with a level of 1024-bit DLOG security [28]. Thus, the elements in G are of 1024 bits. Therefore, the communication overhead of our scheme is much less than the scheme by Zhang *et al*.

Computational overhead: The computational overhead consists of two parts: the generation process and the match phase. We give the comparison results in Table 4, where $H_{G}$ denotes the operation of mapping a bit-string to an element G and *H* represents the hash operation. Obviously, the computational overhead is much less than that in [18].

**Table 4:** The comparison of the computational overhead.

	Scheme in [18]	Our Scheme
Generation phase	(4+n)Exp + $nH_{G}$ + Mul + Pair	5nH
Match phase	2(n+1)Mul + 2Mul + 3Pair	5nH

#### 5.2.2. Implementation

**Figure 6:**
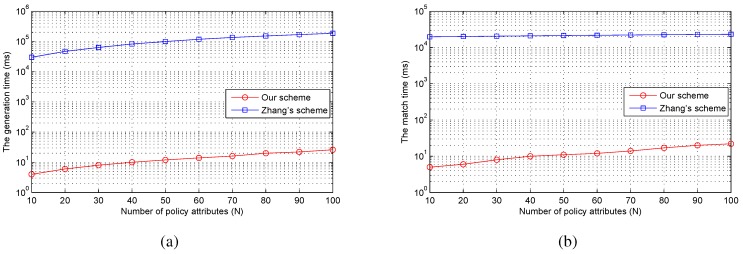
The performanceof match schemes. (**a**) the generation time for matching; (**b**) the match time.

To demonstrate the efficiency of our scheme in practice, we implement our scheme and the scheme in [18] on smartphones with the same platform as before. The number of *n* increases from 1–100. For each access policy, we repeat our implementation 10 times on the smartphone. As Figure 6a shows, the generation time of both schemes increases linearly with the number of policy attributes. The total time is 185.3 s for [18] and 26 ms for our scheme when the number of policy attributes is 100. As for the match time, the time for [18] increases slowly with the number of policy attributes, while the relation between the match time and the number of policy attributes for our scheme is linear. The total match time is 23.1 s for in [18] and 22 ms for our scheme when the number of policy attributes is 100, as shown in Figure 6b. It can be found that our scheme is significantly more efficient than [18]. The reason is that the computation cost of attribute matching detection increases with the number of fundamental cryptographic operations. The work in [18] needs exponentiation and multiplication operations, while our scheme just needs the hash operations. The exponentiation and multiplication operations are much more expensive than the hash operation. Notice that we do not compare the computational overhead of the match process with [15,16]. As in [18], the number of pairing operations for matching detection in [15,16] linearly grows with the number of *n*, and the pairing operation costs much more than the exponentiation and multiplication operations.

## 6. Conclusions

In this paper, we proposed EPPS, which could achieve efficient and privacy-preserving PHI sharing in MHSNs by using ABE. To hide the sensitive access policy, we let each attribute contain an attribute name and its corresponding values, used the Bloom filter to realize partial access policy hidden and conducted the access policy checking before decryption. Moreover, we outsourced most of the time-consuming ABE decryption to the cloud, while preventing the cloud from learning anything about the plaintext and access policy. Through security and performance analysis, we found that our EPPS could achieve fine-grained access control, hidden access polices for PHI sharing and could be easily implemented in resource-constrained mobile devices.
